# Nesprin-2 Interacts with Condensin Component SMC2

**DOI:** 10.1155/2017/8607532

**Published:** 2017-12-27

**Authors:** Xin Xing, Carmen Mroß, Linlin Hao, Martina Munck, Alexandra Herzog, Clara Mohr, C. P. Unnikannan, Pranav Kelkar, Angelika A. Noegel, Ludwig Eichinger, Sascha Neumann

**Affiliations:** ^1^Institute of Biochemistry I, Medical Faculty, University Hospital Cologne, Joseph-Stelzmann-Str. 52, 50931 Cologne, Germany; ^2^Center for Molecular Medicine Cologne (CMMC) and Cologne Cluster on Cellular Stress Responses in Aging-Associated Diseases (CECAD), Medical Faculty, University of Cologne, Cologne, Germany

## Abstract

The nuclear envelope proteins, Nesprins, have been primarily studied during interphase where they function in maintaining nuclear shape, size, and positioning. We analyze here the function of Nesprin-2 in chromatin interactions in interphase and dividing cells. We characterize a region in the rod domain of Nesprin-2 that is predicted as SMC domain (aa 1436–1766). We show that this domain can interact with itself. It furthermore has the capacity to bind to SMC2 and SMC4, the core subunits of condensin. The interaction was observed during all phases of the cell cycle; it was particularly strong during S phase and persisted also during mitosis. Nesprin-2 knockdown did not affect condensin distribution; however we noticed significantly higher numbers of chromatin bridges in Nesprin-2 knockdown cells in anaphase. Thus, Nesprin-2 may have an impact on chromosomes which might be due to its interaction with condensins or to indirect mechanisms provided by its interactions at the nuclear envelope.

## 1. Introduction

The nucleus of a eukaryotic cell harbors the genetic material that is organized in long DNA polymers and is associated with numerous proteins to form chromatin. Chromatin is separated from the cytoplasm by the nuclear envelope (NE), a continuous membrane system consisting of an inner (INM) and an outer nuclear membrane (ONM) enclosing the perinuclear space (PNS). Both membranes are connected at the nuclear pore complexes, the ONM continues into the endoplasmic reticulum (ER). The NE is not a simple membrane barrier but is lined with and crossed by large protein assemblies that provide it with various cellular functions. Nesprins (nuclear envelope spectrin repeat proteins) together with SUN proteins are central components of the NE. Currently four Nesprins (Nesprins-1–4) are known in mammals. They reside at the INM and ONM, have different sizes, and exist in many isoforms [1]. Nesprins are characterized by a varying number of spectrin repeats followed by a C-terminal KASH (Klarsicht, ANC-1, Syne Homology) domain which anchors the proteins in the nuclear membrane and interacts with the SUN domain of SUN proteins in the perinuclear space [2]. Nesprin-1 and Nesprin-2 harbor at their N-terminus paired calponin homology domains that mediate the binding to F-actin [3, 4]. The N-terminus of Nesprin-3 binds to plectin, a cytoskeletal crosslinker that establishes the connection to the intermediate filament system [5]. Nesprin-4 interacts with kinesin-1, a motor protein that uses microtubules as cellular routes [6]. Microtubule interaction through kinesin-1 has also been described for Nesprin-2 [7].

Based on the nucleo-cytoskeletal interactions, Nesprins integrate the nucleus into the cytoskeleton of a cell and participate in the maintenance of nuclear shape and stability [8, 9]. The spectrin repeats (SRs) are platforms for protein-protein or self-interactions [10]. Furthermore, the number of SRs and therefore the length of the rod have been proposed to modulate the size of the nucleus [11]. In the central SR domain, an additional domain has been described in Nesprin-2, an SMC (structural maintenance of chromosomes) domain encompassing amino acid residues 1,464–1,771, which was identified by Dawe et al. [12] as an interaction site for meckelin, a protein with functions in the formation of primary cilia. Primary cilia are sensory organs that act as mechanoreceptors in various signaling pathways or sensors of chemical stimuli [13].

SMC proteins have core functions in regulating genome stability and the organization of the genetic material. They are present from bacteria to man [14]. Classical SMC proteins are composed of 1,000–1,300 amino acids. They have two coiled-coil regions interrupted by a central hinge. The coiled coils fold back on themselves and form an extended structure. At their ends, the N- and C-termini of a molecule interact with each other to form a globular ATP-binding domain [15]. The hinge regions are responsible for heterodimerization of SMC molecules [16]. Six SMC proteins have been described in man, SMC1–6. SMC1/3 form the core of the cohesin complex which mediates sister chromatid cohesion; SMC2/4 are present in the condensin complex that acts in chromosome assembly and segregation. They are present in two condensin complexes with distinct roles, condensins I and II, which contain SMC2 and SMC4 in combination with different non-SMC subunits. Condensins I and II are associated sequentially with chromosomes during the cell cycle and have different roles for chromosome architecture. Condensin I is not present in the nucleus in interphase. During mitosis, condensin I is required for removal of cohesin from chromosome arms and for chromosome shortening, whereas condensin II plays a role in chromosome condensation during early prophase [17]. Condensin I is, however, not completely excluded in interphase from the nucleus since a small pool was found in association with intergenic and intronic regions during interphase [18, 19]. By contrast, condensin II is always nuclear. It is associated with DNA throughout interphase and concentrates on chromosomes in prophase. Based on its interphase distribution, a role in nuclear architecture was proposed [20, 21]. Cohesin and condensin complexes have also roles in DNA repair and gene regulation throughout the cell cycle [20]. Moreover, condensin is involved in organizing the chromatin allowing intrachromosomal associations of gene loci as shown in fission yeast [22]. SMC5/6 is mainly implicated in DNA damage repair and DNA recombination and has specific roles in meiosis [23, 24].

We have carried out a biochemical and functional characterization of the Nesprin-2-SMC domain, hereafter referred to as Nesprin-2-SMC. We show that it can self-assemble to form dimers, trimers, and higher order structures and can interact with condensin proteins SMC2 and SMC4. Monoclonal antibodies directed against the SMC domain showed a distribution of the Nesprin-2 isoforms containing the SMC domain along the NE during interphase and a presence at the chromosomes during mitosis. We also uncovered an impact of Nesprin-2 on mitotic chromosomes that might be mediated by an interaction with the condensin core units SMC2/4.

## 2. Materials and Methods

### 2.1. Cell Culture, Transfection, and Cell Synchronization

HaCaT (human keratinocyte cell line), COS7 (African green monkey kidney fibroblasts), and HeLa (human cervical cancer cells) cells were grown in a humidified atmosphere containing 5% CO_2_ at 37°C in DMEM (high glucose, Life Technologies) supplemented with 10% fetal bovine serum (FBS), 2 mM Glutamine (SIGMA), and 1% penicillin/streptomycin. Cells were transfected as described [11]. To knock down Nesprin-2, HaCaT cells were transfected twice at intervals of 72 h using the Amaxa Nucleofector Kit V Solution (Lonza). The plasmids used for knockdown of Nesprin-2 targeting the N-terminus and the C-terminus (Nesprin-2 N-term shRNA, Ne-2 N-term KD; Nesprin-2 C-term shRNA, Ne-2 C-term KD) as well as the control have been described previously [7]. The newly generated plasmids are described below. For cell cycle synchronization, HaCaT cells were treated with thymidine (2 mM) for 24 h and then with Nocodazole (100 ng/ml) for 12 h or alternatively first with 9 *μ*M RO-3306 (Santa Cruz Biotechnology, sc-358700) for 20 to 22 h and then approximately 3 h release (depending on the desired mitotic phase) to obtain mitotic phases. RO-3306 is a CDK1 inhibitor and reversibly arrests proliferating cells at the G2/M phase of the cell cycle [26]. FACS analysis of cell cycle stages was performed with unsynchronized and synchronized cells. Staining was done with Nuclear-ID™ Red DNA (Enzo ENZ-52406).

Determination of cell proliferation was done by plating at one time point six wells each with the same number of cells and then counting two wells after 24 h, two after 48 h, and two after 72 h.

### 2.2. Cloning Strategies

cDNAs from HaCaT cells encoding the SMC domain in Nesprin-2 (AAN60443, aa 1436–1766, and SRs 11–13) were used as PCR templates using the primers for the following: 5′GAATTCAATGAACTCCTTAAAAATATTCAAGATGTG 3′, rev: 5′GAATTCCTCGAGGGATTCAGTCATCCCGATCTGGGTCTTGG 3′ that contain* EcoRI* restriction sites for cloning into pGEX-4T1 (Amersham) yielding pGEX-4T1-Nesprin-2-SMC which encodes GST-Nesprin-2-SMC. GST is located at the amino terminus of the protein. Nesprin-2-SMC sequences were generated by PCR and cloned into pCMV-Myc (GE Healthcare) using pGEX-4T1 Nesprin-2-SMC as template and primers with* EcoRI* or* XhoI *restriction sites, SMC (SR11–13) for the following: 5′GAATTCTGAATGAACTCCTTAAAAATATTCAAGATGTG 3′, rev: 5′CTCGAGCTAGAGGGATTCAGTCATCCCGATCTGGGTCTT 3′. SR11 for: 5′GAATTCTGAATGAACTCCTTAAAAATATTCAAGATGTG 3′, rev: 5′CTCGAGCTATCTCCCACATTGTTCAAGACATTCGGTGAC 3′, SR12 for: 5′CTCGAGGTTTTGGAGCTCTTAAAACAATATCAGAAT 3′, rev: 5′CTCGAGCTAACCAAGATTTTCATAGTAATCTTCTGTCTT 3′, SR13 for: 5′GAATTCTGCGAGCTCTAGCTTTGTGGGACAAACTTTTTA 3′, rev: 5′CTCGAGCTAGAGGGATTCAGTCATCCCGATCTGGGTCTT 3′. Myc-SR53–56 corresponding to residues 6146–6799 of Nesprin-2 is described in Schneider et al. [7].

A Nesprin-2 SMC domain specific shRNA (Ne-2 SMC) was generated as described using the following oligonucleotides: sense 5′-ATTCTCCTGTTAAGCACTTCTGTACATGGAAGCTTGCATGTATAGGAGTGCTTAGCAGGAGAATCCATTTTTT-3′, antisense 5′-GATCAAAAAATGGATTCTCCTGCTAAGCACTCCTATACATGCAAGCTTCCATGTACAGAAGTGCTTAACAGGAGAATCG-3′ and a random control using sense 5′-CCTTTCAGATACGTCTTGTACAGGTATTGAAGCTTGAATGCCTGTACAGGATGTATCTGAAAGGCGATTTTTT-3′ and antisense 5′GATCAAAAAATCGCCTTTCAGATACATCCTGTACAGGCATTCAAGCTTCAATACCTGTACAAGACGTATCTGAAAGGCG-3′ oligonucleotides [27]. The efficiency of the knockdown was evaluated by immunofluorescence and western blot analysis. Knockdown of SMC2 in COS7 cells was achieved with SMC2-specific siRNAs (E-006836-00-0005, Dharmacon, GE Healthcare). For control, corresponding scrambled shRNA was used. The cell line was recommended by the supplier in combination with the particular siRNAs. Transfection was carried out using Dharmafect transfection reagent according to the manufacturer's protocol. The cells were analyzed 96 h after the transfection. Successful knockdown was assessed by immunofluorescence analysis using SMC2 specific antibodies.

### 2.3. Expression and Purification of GST Proteins and GST Pulldown

Plasmids encoding GST fusion proteins were transformed into* E. coli* XL-1 blue and grown overnight and diluted 1 : 50 into fresh LB media. The bacteria were grown to an OD_600_ of 0.6 to 0.8 when they were induced with 0.5 mM IPTG and the protein expression was continued overnight at 20°C. Bacteria were pelleted and washed with STE buffer (10 mM Tris-HCl, pH 8.0, 50 mM NaCl, and 1 mM EDTA). Lysis was achieved by the addition of 100 *μ*g/ml lysozyme and mechanical shearing in a Dounce homogenizer followed by centrifugation. Fusion proteins were bound to Glutathione-Sepharose 4B (GE Healthcare). The GST-Nesprin-2-SMC polypeptide has a predicted molecular weight of 64.8 kDa. It was efficiently expressed in* E. coli* XL-1 blue and purified as soluble proteins. The protein was bound to Glutathione-Sepharose beads and Nesprin-2-SMC was released from the GST part by thrombin cleavage (Sigma-Aldrich). Alternatively, GST-Nesprin-2-SMC was eluted from the beads with reduced glutathione (20 mM) in 100 mM Tris-HCl, pH 8.0.

GST pulldown assays were performed by lysing HaCaT or COS7 cells in lysis buffer (50 mM Tris-HCl, pH 7.5, 150 mM NaCl, 1% Nonidet P-40, and 0.5% sodium deoxycholate) supplemented with protease inhibitor cocktail (Sigma-Aldrich) by pushing them through a 0.4 mm needle followed by sonication and centrifugation. Cell lysates were incubated with Glutathione-Sepharose beads overnight for binding to the GST fusion proteins or GST and washed 5 times with PBS or lysis buffer supplemented with protease inhibitors. Beads bound protein complexes were analyzed by SDS-PAGE and western blot (WB).

### 2.4. Antibodies and Immunofluorescence (IF) Microscopy

The following antibodies were used: mouse monoclonal anti-Nesprin-2 mAb K20-478 raised against the actin binding domain (ABD) of Nesprin-2 (residues 1–285) [3] (IF, 1 : 200; hybridoma supernatant, WB, 1 : 10), rabbit polyclonal antibodies pAbK1 raised against spectrin repeats in the C-terminal region of Nesprin-2 [28] (IF, 1 : 100; WB, 1 : 1,000), Nesprin-1 specific mAb K43-322-2 raised against N-terminal spectrin repeats 10 and 11 of Nesprin-1 [29] (hybridoma supernatant, undiluted), GFP-specific mAb K3-184-2 [30] (hybridoma supernatant, IF, 1 : 2; WB, 1 : 10), Myc-specific mAb 9E10 [31] (hybridoma supernatant, IF, undiluted; WB, 1 : 10), pAb against GST [32] (WB, 1 : 50,000), mAb K84-913 against GST (hybridoma supernatant, WB 1 : 10), pAb Lamin B1 (Abcam ab16048, IF, 1 : 200; WB, 1 : 4,000), pAb SMC2 (Novus Biologicals NB100-373, IF, 1 : 100; WB, 1 : 2,000), WB: mAb SMC4 (Abcam ab179803 1 : 2,000), IF: pAb SMC4 (Abcam ab17958, 1 : 500), pAb SMC1 (Abcam ab21583, WB 1 : 1000), goat SMC3 (Santa Cruz Biotechnology, sc-8135, WB 1 : 50), rabbit CAP-H (Biomol-Bethyl A300-603A-T, WB 1 : 1000), pAb CAP-H2 (Biomol-Bethyl A302-275A, WB 1 : 4000), mAb PDI (Abcam ab2792, 1 : 100), pAb calreticulin (Thermo Fisher PA3-900, IF 1 : 50–200), and rat mAb YL1/2 specific for *α*-tubulin (1 : 5). mAb K81-116-6 (hybridoma supernatant, undiluted) directed against the SMC domain in Nesprin-2 was generated in this study. The antibodies were used for immunofluorescence and western blot analysis. A polypeptide corresponding to Nesprin-2 aa 1436–1766 (calculated molecular weight 38.78 kDa) was produced as GST fusion polypeptide and bound to Glutathione-Sepharose beads as described above. The SMC polypeptide was liberated by thrombin cleavage and used for production of monoclonal antibodies by immunization of mice as described [33]. Alexa 568 or 488 fluorescently labeled and highly cross absorbed and affinity purified secondary antibodies were used (Thermo Fisher), and 4,6-diamino-2-phenylindole (DAPI, Sigma) was used to visualize DNA. For immunofluorescence, cells grown on cover slips were fixed in 3% paraformaldehyde (PFA) in phosphate-buffered saline (PBS) for 15 min followed by 4 min incubation with 0.5% Triton X-100/PBS. Alternatively, cells were fixed by 10 min incubation in ice cold methanol at −20°C. Blocking was done with PBG (0.5% BSA, 0.045% fish gelatine in PBS, pH 7.4) at room temperature (RT) for 30 min. Primary and secondary antibodies as well as DAPI were diluted in PBG and applied to the cells for 1 h at RT or overnight at 4°C. Microscopy was performed by using TCS-SP5 (Leica) or the Ångstrom Opti Grid confocal microscope (Leica). For control, cells were routinely labeled with secondary antibodies only. In no case was a signal obtained.

To test the specificity of the newly established mAb K81-116-6, the antibodies were removed from the hybridoma supernatant (depletion) and the supernatant was then used for immunofluorescence analysis. Depletion was performed in two ways. For one, the hybridoma supernatant was incubated with Glutathione-Sepharose beads carrying GST-Nesprin-2-SMC polypeptides. The beads were removed by centrifugation (2000 rpm, 2 min) and the supernatant was used for immunofluorescence analysis. Alternatively, GST-Nesprin-2-SMC was loaded onto a SDS-polyacrylamide gel, the protein was then transferred to a nitrocellulose membrane, detected by Ponceau S staining, and the part of the membrane carrying GST-Nesprin-2-SMC protein was cut out and incubated with mAb K81-116-6. After overnight incubation (4°C), the solution was removed from the membrane and applied for IF. For both approaches, an aliquot of the antibody solution before depletion was kept for control.

### 2.5. Immunoprecipitation

For immunoprecipitation (IP), HaCaT cells were harvested and lysed in lysis buffer (50 mM Tris-HCl, pH 7.5, 150 mM NaCl, 1% Nonidet P-40, 0.5% sodium deoxycholate, and protease inhibitor cocktail). Cells were lysed by pushing and pulling through a 0.4 mm needle and centrifuged (12.000 rpm, 20 min). Supernatants were incubated for 1 h with protein A Sepharose CL-4B beads (GE Healthcare) for preclearing. Subsequently, beads were removed by centrifugation (2000 rpm, 2 min) and cell lysates incubated with 5–8 *μ*g of the antibody of interest for 2 h at RT. Protein A Sepharose CL-4B beads equilibrated with lysis buffer were then added to the cell lysates and incubation was continued overnight at 4°C. The beads were collected by centrifugation and washed five times with PBS and the bound proteins released from the beads by addition of SDS sample buffer and heating to 95°C for 5 min and analyzed by SDS-PAGE (3–12% acrylamide for gradient gels; 10% and 12% acrylamide as appropriate) and western blotting. Transfer of high molecular weight Nesprin-2 giant to nitrocellulose membranes (0.22 *μ*m pore size) was done by wet blotting technique for two to three days.

### 2.6. Gel Filtration and Chemical Cross-Linking

To assess the oligomeric state of the native protein, the sample was applied to a gel filtration column (Sephadex G-200, GE Healthcare) as described [34]. For molecular weight determination, molecular weight standards (GE Healthcare) were separated under identical conditions. Chemical cross-linking of Nesprin-2-SMC (1 mg/ml) was performed with the zero-length cross-linking reagent EDC (1-ethyl-3-[3-dimethylaminopropyl]carbodiimide hydrochloride) (Thermo Fisher) together with sNHS (Sulfo-*N-*hydroxysuccinimide) in 0.1 M MES buffer (pH 6.5) [35].

## 3. Results

### 3.1. Nesprin-2 Contains an SMC Domain in Its Rod Domain

**Figure 1:**
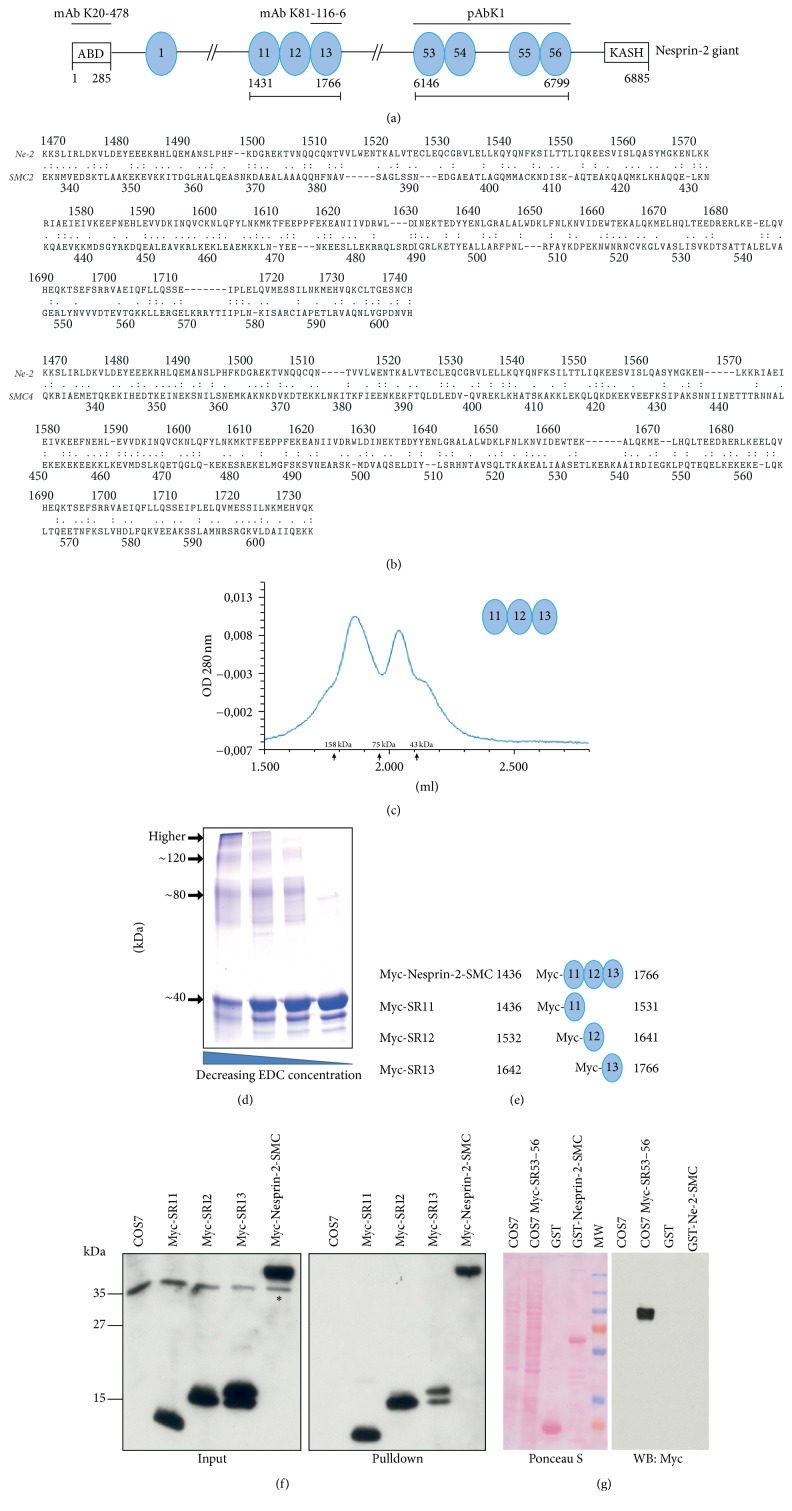
Characterization of the SMC domain of Nesprin-2. (a) Schematic of Nesprin-2 (not drawn to scale). The location of the SMC domain (spectrin repeats 11–13) and the C-terminal spectrin repeats (53–56) is shown. Epitopes of antibodies used are indicated above the schematic. ABD, actin binding domain; ovals, spectrin repeats. The spectrin repeat domain starts at position 308. (b) Sequence comparison of the Nesprin-2-SMC domain with coiled-coil regions of SMC2 and SMC4. The sequence comparison was performed using LALIGN, the Pairwise Sequence Alignment tool from EMBL-EBI (https://www.ebi.ac.uk/Tools/psa/lalign/). Nesprin-2 (NCBI GenBank accession number AF435011.1), SMC2 (NCBI GenBank accession number O95347.2), and SMC4 (NCBI GenBank accession number Q8WXH0.3) were used.  :, identical amino acid;  ., conservative substitution. (c) Analysis of Nesprin-2-SMC by gel filtration chromatography. UV traces of the elution profile are shown. Nesprin-2 SMC (calculated molecular weight 39 kDa). Molecular weight markers were ovalbumin (43 kDa), conalbumin (75 kDa), and aldolase (158 kDa). (d) Analysis of chemically crosslinked Nesprin-2-SMC. Zero-length cross-linking reagent EDC (1-ethyl-3-[3-dimethylaminopropyl] carbodiimide hydrochloride) was used at decreasing concentrations. The proteins were separated by SDS-PAGE (10% acrylamide) and stained with Coomassie Blue. (e) Schematic representation of Myc-tagged Nesprin-2-SMC polypeptides. Amino acid positions refer to human Nesprin-2 giant (accession number AF435011.1). (f) Interaction of GST-Nesprin-2-SMC with individual Myc-tagged spectrin repeats derived from Nesprin-2-SMC and expressed in COS7 cells. GST-Nesprin-2-SMC was used for pulldown (right panel). Western blots were probed with mAb 9E10 specific for Myc. Asterisk, endogenous Myc [25]. (g) Specificity of the Nesprin-2-SMC interaction. Myc-SR53–56 expressed in COS7 cells was used for pulldowns with GST for control and GST-Nesprin-2-SMC. COS7 and COS7 Myc-SR53–56 represent whole cell lysates. The Ponceau S stained blot and the corresponding blot probed with mAb 9E10 are shown. MW, molecular weight marker (from top to bottom: 200, 130, 100, 70, 55, 35, and 25 kDa).

We investigate here a region in the SR containing rod domain of Nesprin-2 with homology to the SMC (Structural Maintenance of Chromosomes) domain (*E* value 9.34*e* − 0.3). This domain encompasses amino acids 1436–1766 and extends over SR11–13 designated Nesprin-2-SMC (Figure 1(a)) [36]. In a comparison with mammalian SMC proteins, we found high degrees of homology with the coiled-coil regions of SMC2 and SMC4 (19.7% identity, 52.9% similarity and 21.5% identity, 53.9% similarity, resp.) (Figure 1(b)). To assess whether Nesprin-2-SMC can undergo self-interactions, we expressed it as GST fusion protein and analyzed the elution behavior of the 39 kDa polypeptide, which had been released from GST by thrombin cleavage, by size exclusion chromatography. The protein eluted in two peaks, one eluting at ~50 kDa and corresponding to the monomer and a broader and larger one eluting between 75 kDa and 158 kDa indicative of oligomers (Figure 1(c)). The proteins used for calibrating the column are globular proteins, whereas Nesprin-2-SMC is expected to be a rod shaped molecule presumably affecting the elution behavior. The elution pattern was also confirmed by SDS-PAGE and staining with Coomassie Blue which showed that the protein eluted in fractions in front of ovalbumin indicating an oligomeric state (Figure S1(a)). Cross-linking experiments using varying concentrations of the zero-length cross-linking reagent EDC showed the presence of monomers, dimers, trimers, and even higher molecular weight complexes. With decreasing EDC concentration, the amount of higher molecular weight forms decreased whereas the monomeric form increased (Figure 1(d)). The oligomerization property of Nesprin-2-SMC was supported by data from pulldown experiments in which GST-Nesprin-2-SMC precipitated Nesprin-2 giant from HaCaT cell lysates (see Materials and Methods for experimental details). Human Nesprin-2 giant is a 6,885-amino-acid protein with a predicted molecular weight of 796 kDa. Mass spectrometric analysis identified peptides covering the entire Nesprin-2 giant molecule in the precipitate (Figure S1(b)). The high coverage of the sequence located between residues 1436 and 1766 was due to the polypeptide used for the pulldown. GST did not precipitate Nesprin-2.

We further expressed Myc-tagged Nesprin-2-SMC (Myc-Nesprin-2-SMC) corresponding to the full length SMC domain of Nesprin-2 and Myc-tagged polypeptides corresponding to its individual SR domains in COS7 cells and used the cell lysates for pulldown experiments with GST-Nesprin-2-SMC (Figure 1(e)). GST-Nesprin-2-SMC precipitated Myc-Nesprin-2-SMC and its individual SRs from COS7 cell lysates as shown in the immunoblot using Myc-specific antibody mAb 9E10 (Figure 1(f)). Taken together, the results suggest that the Nesprin-2-SMC domain has the potential to oligomerize. We then asked whether this interaction is specific to this Nesprin-2 domain and tested whether GST-Nesprin-2-SMC could interact with other spectrin repeats of Nesprin-2. We therefore expressed Myc-SR53–56 composed of the last four spectrin repeats of Nesprin-2 (SR53–SR56, aa 6116–6799, Figure 1(a)) in COS7 cells and carried out pulldown assays with GST for control and GST-Nesprin-2-SMC [7]. GST-Nesprin-2-SMC did not precipitate Myc-SR53–56 underlining the specificity of the interaction (Figure 1(g)).

### 3.2. Monoclonal Nesprin-2-SMC Domain Specific Antibodies Detect a High Molecular Weight Protein and Stain the Nuclear Envelope

**Figure 2:**
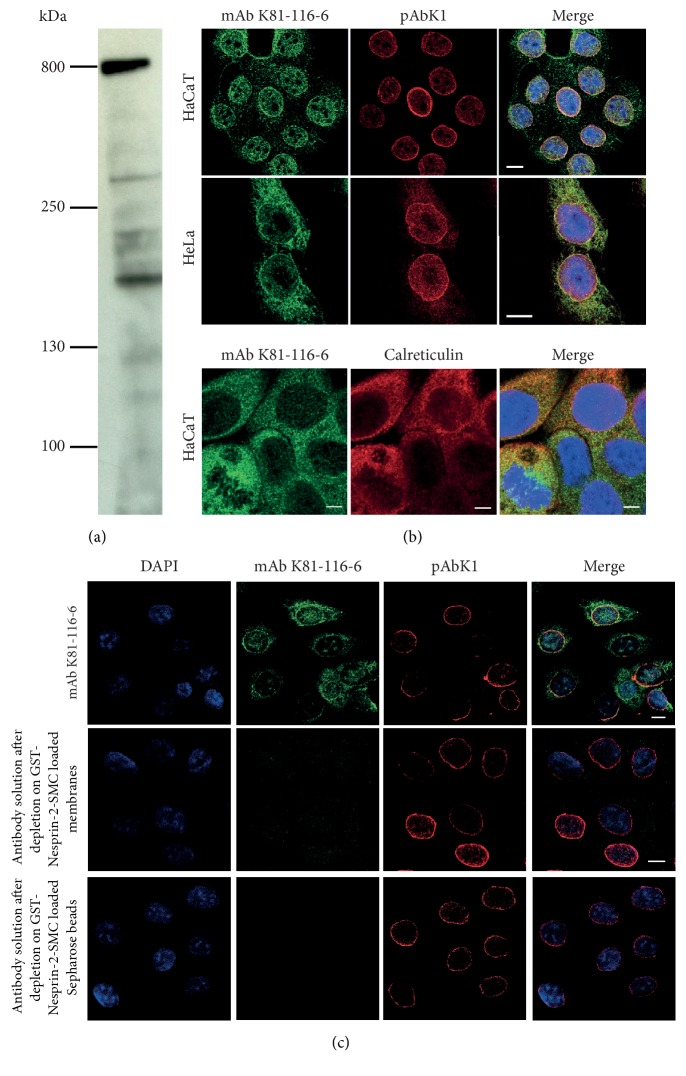
Characterization of monoclonal antibodies directed against the SMC domain. (a) Detection of Nesprin-2 with mAb K81-116-6 in HaCaT cell lysates. Proteins were separated by SDS-PAGE (3–12% acrylamide). (b) mAb K81-116-6 staining of HaCaT and HeLa cells. pAbK1 was used as bona fide Nesprin-2 antibody. DAPI stains the DNA (in Merge). Bar, 10 *μ*m. Lower panel, colocalization of Nesprin-2 detected by mAb K81-116-6 with ER marker calreticulin in HaCaT cells. Bar, 5 *μ*m. (c) Analysis of the specificity of mAb K81-116-6. Antibodies were depleted from the hybridoma supernatant by the indicated procedures. Antibody depleted supernatants were then used for immunofluorescence analysis. Bar, 10 *μ*m.

To study Nesprin-2 isoforms harboring the SMC domain, we generated monoclonal antibodies by immunizing mice with Nesprin-2-SMC polypeptide that had been released from the GST part by thrombin cleavage. In western blots of HaCaT cell homogenates that had been separated in gradient gels (3–12% acrylamide) mAb K81-116-6 recognized primarily a high molecular weight protein which we presume corresponds to the ~800 kDa Nesprin-2 giant [3]. Faint bands below could be degradation products or N-terminal isoforms [1] (Figure 2(a)). In independent experiments, in which we immunoprecipitated Nesprin-2 from HaCaT cells and probed the precipitate with SMC2 and SMC4 antibodies, we excluded that any of the lower molecular weight bands corresponded to SMC proteins due to cross reactivity of the antibodies (data not shown). In immunofluorescence analysis, mAb K81-116-6 labeled the NE in HaCaT and HeLa cells overlapping with the pAbK1 staining (Figure 2(b)). The previously characterized pAbK1 polyclonal antibodies had been generated against the four C-terminal spectrin repeats of Nesprin-2 and are specific for Nesprin-2 (Figure 1(a)) [28]. In addition, mAb K81-116-6 stained structures in the cytoplasm in the vicinity of the nucleus which are possibly membranes of the endoplasmic reticulum (ER) as we observed colocalization with calreticulin, an ER protein (Figure 2(b), lower panel). The cytoplasmic staining was comparatively faint in HaCaT cells, whereas in HeLa cells it was more pronounced. pAbK1 also stained these structures; however the staining was less intense which might be due to different accessibility of the epitopes (Figure 2(b)). Nesprin-2 is a tail-anchored protein and its mRNA has been found anchored to the ER where it is translated. This might explain the observed localization [37].

To prove the specificity of mAb K81-116-6, we carried out antibody depletion studies. We found that the staining of the NE as well as the cytoplasmic staining was completely abrogated after depletion of mAb K81-116-6 from the hybridoma supernatant by incubating the supernatant with nitrocellulose membrane strips carrying GST-Nesprin-2-SMC or with Glutathione-Sepharose 4B beads carrying GST-Nesprin-2-SMC. By contrast, the NE was still labeled by pAbK1 (Figure 2(c)). Furthermore, the protein was no longer detected in cell lysates after knocking down Nesprin-2 using shRNA directed against the SMC domain (Figure S2(a)) and no signals were detected when cells were analyzed by immunofluorescence (see below, Figures 4(b) and 4(c)).

### 3.3. SMC2 Is a Nesprin-2 Binding Partner

**Figure 3:**
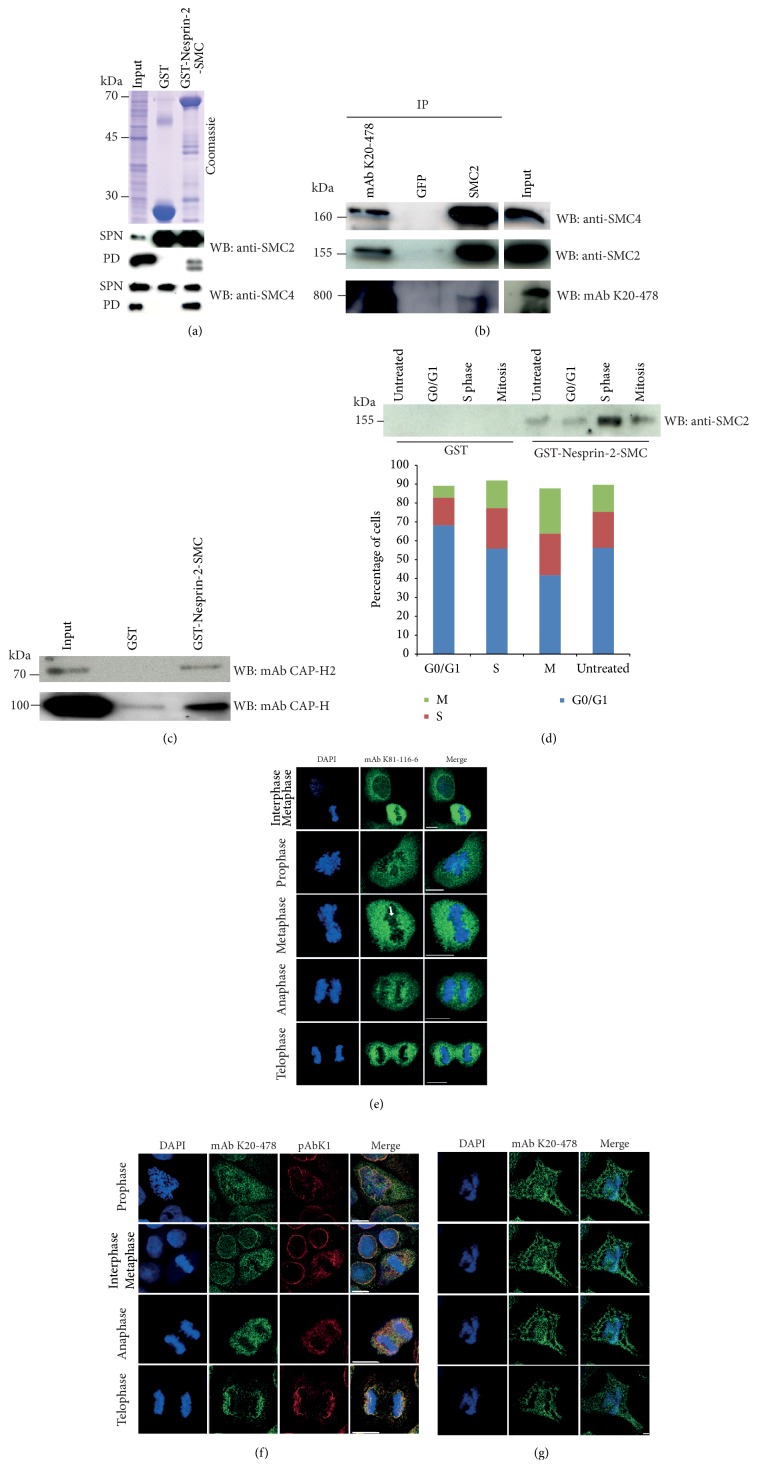
Interaction of Nesprin-2-SMC and Nesprin-2 with SMC2 and SMC4. (a) Precipitation of SMC2 and SMC4 with GST-Nesprin-2-SMC from HaCaT cell lysates. Precipitates were resolved on SDS-polyacrylamide gels (10% acrylamide) and probed with SMC2 and SMC4 specific antibodies. SPN, supernatant after pulldown; PD, pulldown. The lower molecular weight band in the SMC2 pulldown is presumably a breakdown product. (b) Immunoprecipitation of SMC2 from HaCaT cell lysates with Nesprin-2 specific mAbK20-478 and of Nesprin-2 with SMC2 specific antibodies. GFP-specific monoclonal antibodies were used for control. The antibodies used for immunoprecipitation are indicated above the panels (IP). The blots were probed with the antibodies listed on the right (WB). Immunoprecipitates were resolved on gradient gels (3–12% acrylamide) and 10% acrylamide gels as appropriate. The data are from one blot; however, the input was not directly adjacent to the SMC2 IP. (c) Interaction of CAP-H2 (condensin II) and CAP-H (condensin I) with Nesprin-2-SMC. Pulldowns were performed with HaCaT cell lysates and GST for control and GST-Nesprin-2-SMC as indicated. Unsynchronized cells were used for the experiments shown in (a)–(c). (d) Analysis of the Nesprin-2-SMC interaction with SMC2 during the cell cycle. HaCaT cells were synchronized with RO-3306 or other reagents as described in Materials and Methods in order to obtain the relevant cell cycle phases. Cell cycle phases were assessed by FACS analysis; the results are depicted in the accompanying diagram. Pulldown was carried out with GST-Nesprin-2-SMC bound to GST-Sepharose. GST was used for control. The blot was probed with SMC2 specific antibodies. (e) Localization of Nesprin-2 as detected with mAb K81-116-6 (green) during mitosis in HaCaT cells. DNA was stained with DAPI. Arrow points to filamentous staining across the chromosomes. (f) Nesprin-2 distribution in HaCaT cells during mitosis as detected with mAb K20-478 (green) and pAbK1 (red). DNA was detected with DAPI. Bar, 10 *μ*m. (g) Nesprin-2 presence on chromosomes. Different Z-stacks (from top to bottom: 0 *μ*m, 0.21 *μ*m 0.42 *μ*m, and 0.84 *μ*m) from a COS7 cell in anaphase stained with mAb K20-478. DNA was stained with DAPI. Bar, 5 *μ*m.

To identify binding partners for Nesprin-2, we performed immunoprecipitation experiments using mAb K20-478 directed against the N-terminus of Nesprin-2 and pAbK1 (Figure 1(a)). The proteins were separated by SDS-PAGE and stained with Coomassie Blue, bands were cut out, and the proteins were identified by mass spectrometry. For control GFP-specific antibody mAb K3-184-2 was used. Among the precipitated proteins were histones, SUN1, Lamin A/C, and SMC2 which were found in the immunoprecipitate of mAb K20-478. The SUN1 and Lamin A/C interactions have been previously described and are well characterized; the histone and SMC2 interactions are novel findings [2, 28, 38]. Here we followed up the SMC2 interaction. Because of the SMC homology in Nesprin-2, we speculated that this domain could interact with SMC2 and carried out pulldown assays with Glutathione-Sepharose 4B beads loaded with GST-Nesprin-2-SMC using HaCaT cell lysates as described in Materials and Methods and probed the pulldown for the presence of SMC2. GST loaded beads served as control. We could indeed detect SMC2 in the GST-Nesprin-2-SMC precipitate by SMC2 specific antibodies. SMC4 which forms a complex with SMC2 in condensin was also pulled down by GST-Nesprin-2-SMC. GST did not precipitate SMC2 or SMC4 (Figure 3(a)). Further proof for an interaction came from immunoprecipitation experiments from HaCaT cells with mAb K20-478 to precipitate Nesprin-2. In the Nesprin-2 pulldown, we detected SMC2 and SMC4. In the reverse experiment using SMC2 specific antibodies, Nesprin-2 was detected in the precipitate with mAb K20-478. GFP antibodies used for control did not bring down any of the proteins tested (Figure 3(b)).

As condensin exists in two complexes, condensin I and condensin II [18], we used CAP-H (kleisin *γ*, non-SMC condensin I complex subunit H) and CAP-H2 (kleisin *β*, non-SMC condensin II complex subunit H2) antibodies to probe the GST-Nesprin-2-SMC pulldown and identified CAP-H and CAP-H2 in the precipitate (Figure 3(c)). We also probed whether other SMC proteins interacted with Nesprin-2. However, the cohesin components SMC1 and SMC3 were not seen in the precipitate after carrying out a pulldown with GST-Nesprin-2-SMC (Figure S2(b)). These results make the interaction a specific one between condensin and Nesprin-2. Although SMC proteins are present in all phases of the cell cycle, they have specific roles in specific phases [17]. To find out whether the interaction is confined to a particular stage of the cell cycle, we used lysates from HaCaT cells that had been treated with various reagents as described in Materials and Methods. This led to the enrichment of cells in particular cell cycle stages. Pulldown assays were carried out with GST-Nesprin-2-SMC and GST loaded Glutathione-Sepharose beads and the precipitates probed for the presence of SMC2. SMC2 was present in the precipitates obtained from lysates of untreated cells, cells in G0/G1, and from cell samples enriched for S and M phase. The signal was most prominent in lysates from S phase enriched cells followed by M phase cells. The GST-control did not bring down SMC2 (Figure 3(d)). The cell cycle stages were controlled by FACS analysis (Figure 3(d), bar graph).

A colocalization of SMC2 and SMC4 with Nesprin-2 was difficult to visualize at the immunofluorescence level because of the very strong signals for SMC2 and SMC4. However, some overlap indicating a colocalization could be seen particularly in telophase (see below, Figures 5(a) and 5(b), upper panels; see telophases of control cells for overlap).

### 3.4. Nesprin-2 Localization during Mitosis

For studying Nesprin-2 localization during mitosis, we performed immunofluorescence analysis using mAb K81-116-6, mAb K20-478, and pAbK1 (Figures 3(e), 3(f), and 3(g)). All antibodies showed that Nesprin-2 relocated to the cytoplasm upon nuclear envelope breakdown where it colocalized with the ER as revealed by costaining with an antibody specific for the ER marker PDI (protein disulfide isomerase) (Figure S3). It also still surrounded the condensed chromosomes, and Nesprin-2 positive structures extended across the chromosomes in all mitotic phases (Figures 3(e), 3(f), 3(g) and S4). Serial sections through the chromosomes of a mitotic cell confirmed the distribution of Nesprin-2 (Figure 3(g)). At the beginning of anaphase until telophase, we found signals at opposing ends of the dividing chromosome material presumably showing the reformation of the NE (Figure 3(f)). This localization was specific for Nesprin-2 as staining for Nesprin-1 with mAb K43-322-2 did not reveal an association with the chromosomes (Figure S5).

### 3.5. Nesprin-2 Knockdown Does Not Affect Condensin Distribution

**Figure 4:**
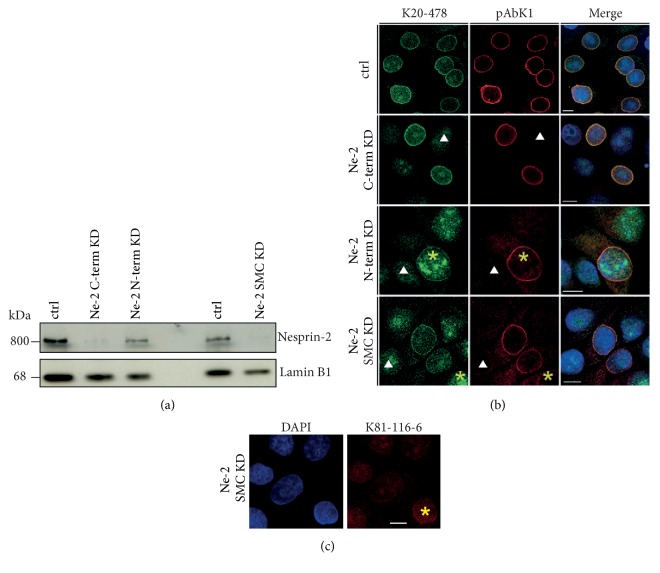
Knockdown of Nesprin-2 using shRNA directed against C-terminal, N-terminal, and SMC domain sequences. (a) Western blots showing the efficiency of the shRNA treatment at the protein level. HaCaT cells were transfected with shRNAs targeting the various regions and for control (ctrl) with the corresponding scrambled shRNAs. Nesprin-2 at ~800 kDa was detected by mAb K20-478. Lamin B1 was used for loading control. (b) Immunofluorescence analysis of HaCaT cells treated with shRNAs targeting the C-terminus (Ne-2 C-term KD), the N-terminus (Ne-2 N-term KD), or the SMC domain (Ne-2 SMC KD). Cells were stained with antibodies directed against the N-terminus (mAb K20-478, green) and the C-terminus (pAbK1, red) of Nesprin-2. DAPI was used to visualize DNA. Arrowhead indicates cells with successful knockdown; asterisk indicates cells which still express Nesprin-2. Bar, 10 *μ*m. (c) Immunolabelling of Ne-2 SMC KD cells with mAb K81-116-6. Nuclei were labeled with DAPI. Asterisk indicates a cell which still expresses Nesprin-2. Bar, 10 *μ*M.

**Figure 5:**
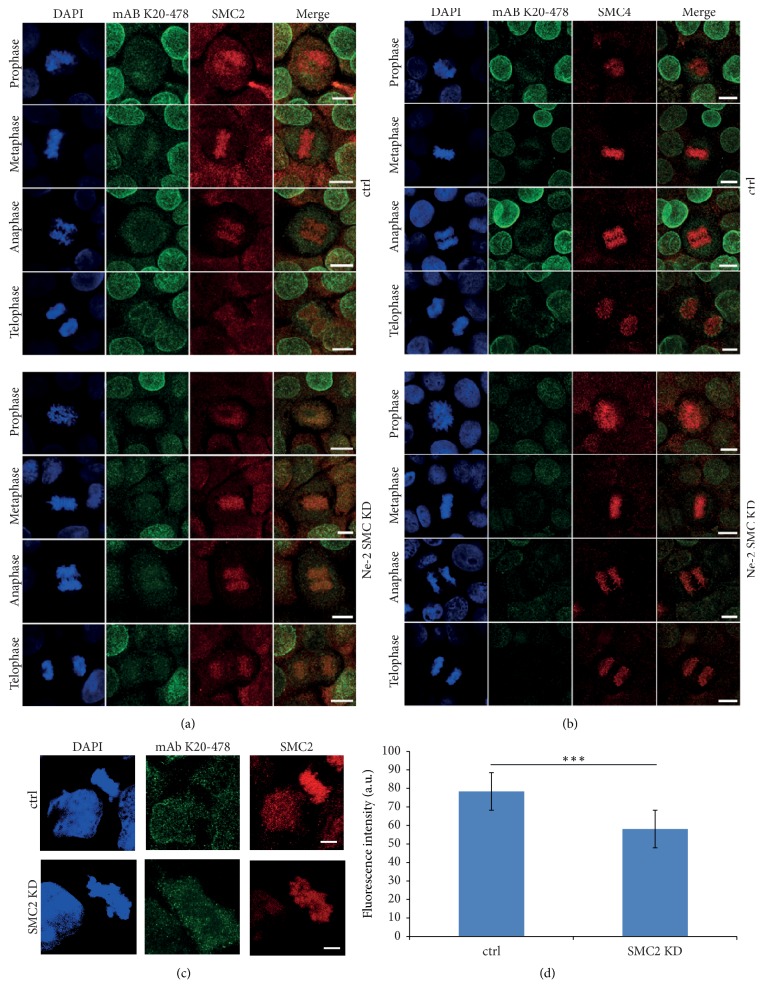
SMC2 (a) and SMC4 (b) in HaCaT keratinocytes treated with control shRNA (upper panels) and treated with Nesprin-2-SMC domain specific shRNA (lower panels). Nesprin-2 was detected with mAb K20-478. Bar, 10 *μ*m. (c) Localization of Nesprin-2 after siRNA mediated knockdown of SMC2 in COS7 cells. Staining was with SMC2 specific antibodies and mAb K20-478 for Nesprin-2. Bar, 5 *μ*m. (d) Evaluation of the SMC2 knockdown. SMC2 fluorescence intensity was measured in the center of mitotic chromosomes. 10 siRNA treated cells and 12 control cells (control treatment) were analyzed (^*∗∗∗*^*P* value = 0.0001).

To specifically explore the role of SMC domain containing Nesprin-2 isoforms, HaCaT cells were treated with Nesprin-2-SMC shRNAs (Ne-2 SMC KD) and compared to cells treated with shRNAs targeting the Nesprin-2 N-terminus or the Nesprin-2 C-terminus (Ne-2 N-term KD; Ne-2 C-term KD) [7]. The sequences for the generation of the SMC-specific shRNAs were carefully chosen in order to exclude off-target effects due to homology to SMC sequences. In western blots labeling with mAb K20-478 revealed a strong reduction of Nesprin-2 giant at ~800 kDa in lysates from cells treated with Ne-2 C-term and Ne-2 SMC shRNAs (Figure 4(a)). Similar results were obtained with mAb K81-116-6 (see above and Figure S2(a)). The knockdown was confirmed at the immunofluorescence level with mAb K20-478, pAbK1, and mAb K81-116-6 (Figures 4(b) and 4(c)). Cell proliferation was not altered in the knockdown cells as compared to HaCaT control cells (two independent experiments, Figure S6(a)). Similarly, FACS analysis did not reveal changes in the progression through the cell cycle (three independent experiments, Figure S6(b)). Nesprin-2 depletion using Ne-2 SMC shRNA did not have an obvious effect on SMC2/4 location as the staining in immunofluorescence analysis was comparable to control cells. Also, SMC2/4 distribution during mitosis was not affected and the proteins had an apparently unaltered association with mitotic chromosomes at the level of analysis (Figures 5(a) and 5(b)). Furthermore, the protein levels appeared unaltered (Figure S2(c)).

We also performed the converse experiment by downregulating SMC2 in COS7 cells by transfection with a siRNA pool targeting SMC2. Since the knockdown was not complete, we searched for mitotic cells with reduced SMC2 staining and analyzed the Nesprin-2 distribution. We found that Nesprin-2 still surrounded the chromosomal mass indicating that Nesprin-2 localization is not strictly dependent on SMC2 (Figures 5(c) and 5(d)).

However, the analyses of the Nesprin-2 depleted cells revealed the presence of chromatin bridges during ana- and telophase. When we determined the chromatin bridges in cells transfected with SMC control and Ne-2 SMC shRNA at ana- and telophase, we observed that 4.4% (mean value) of control cells harbored chromatin bridges. In the Nesprin-2 knockdown cells, this number was increased to 10.3% (*P* value, 0.01; 440 and 544 ana- and telophases evaluated, resp.). This is a Nesprin-2 specific result as the Ne-2 N-term KD also led to enhanced chromatin bridge formation (15.25%, 445 ana- and telophases evaluated). Increased number of chromatin bridges in anaphase has been described for condensin II knockout cells as well as condensins I and II depleted cells [39, 40].

## 4. Discussion

Research on the Nesprins primarily focuses on the interphase nucleus and their role in nuclear positioning, maintaining mechanical and structural properties of the nucleus and the perinuclear cytoskeleton, and their role in signal transduction [1, 41, 42]. We found that during mitosis Nesprin-2 was present along mitotic condensed DNA. In previous studies, we reported that Nesprin-2 interacts with chromatin; in particular centromeric and other heterochromatic reads were enriched in the ChIP-seq data [9]. However, the nature of this interaction is unclear and it might well be an indirect one since Nesprin-2 interacts with proteins present in the chromatin such as histones or SMC proteins. We focused here specifically on the interaction with SMC proteins. In open mitosis, the NE breakdown (NEBD) starts during prophase resulting in a removal of the NE from chromatin. We found that Nesprin-2 was still associated with mitotic chromosomes and Nesprin-2 knockdown cells harbored increased numbers of chromatin bridges in anaphase cells.

In vertebrates, condensins I and II are both composed of the SMC2/4 heterodimer together with distinct additional non-SMC subunits, CAP-G/G2, CAP-D2/D3, and CAP-H/H2 [18]. A depletion of condensin I or II or a combination of both in HeLa cells led to delayed chromosome condensation and caused segregation problems resulting in cells with bridged or lagging chromosomes [17, 41]. In mouse embryonic stem cells, RNA interference studies revealed that condensins I and II are required for ES cell proliferation and that their loss leads to delayed initiation of anaphase and formation of enlarged and misshapen interphase nuclei [43]. Altered nuclear architecture and size after condensin II knockdown were also described more recently [44].

Since we propose a role for Nesprin-2 on chromosomes and also on mitotic chromosomes, we searched publications reporting chromatin proteomes for the presence of Nesprin-2. Nesprin-2 was present in interphase chromatin [45] where it was listed in the category “non-expected chromatin function,” and Nesprin-2 peptides were also identified in a report on nascent chromatin capture proteomics [46]. By contrast, in a publication describing the mitotic proteome, only Nesprin-1 was listed [47]. Taken together, data from independent proteomic approaches support our findings on the presence of Nesprin-2 on chromatin.

Based on the well-known structure and assembly of SMC monomers into pentameric ring complexes, it appears unlikely that the predicted SMC domain in Nesprin-2 fulfills the role of a classical SMC protein. SMC proteins form heterodimers and each dimer consists of a single polypeptide that follows a V-shaped topology. SMC monomers are connected along the hinge region and the terminal ends form catalytically active ATPases [16]. Currently, no Nesprin-2 isoform has been described that might exist as a separate isoform composed of the SMC domain only [48]. It might rather be that the SMC domain in Nesprin-2 interacts with SMC2/4 along their coiled coils. Alternatively, the interaction between condensin and Nesprin-2 is an indirect one. Interestingly, Nesprin-2 knockdown does not have an effect on mitotic progression but preliminary data indicate that the chromosomes in metaphase cells have a fuzzy appearance and a larger volume [49, 50]. Similar observations were made after SMC knockdown and this observation could place Nesprin-2 in this pathway [51]. In this context, Nesprin-2 might adopt a role similar to the one previously suggested for NE proteins in transcriptional regulation where they are thought to regulate the spatiotemporal accessibility of transcriptional regulators to their nuclear targets instead of directly acting as transcriptional regulators in the proximity of genes [52, 53]. Nesprin-2 might act on SMC2/4 in a similar way. Our data indicate that a loss of Nesprin did not prevent SMC2/4 proteins to assemble along mitotic chromosomes but an increased number of chromatin bridges were observed which hints at changes in the process of chromosome separation. It could therefore well be that Nesprin-2 affects directly or indirectly the spatiotemporal assembly or the function of SMC proteins along chromosomes.

In our analysis, we observed that the condensin Nesprin-2 interaction occurred throughout the cell cycle. Interestingly, condensins have roles not only during mitosis but also in interphase, where they are important particularly in gene regulation. For instance, a function in transcriptional regulation has been reported for condensins I and II by Li et al. [19] who found them on enhancers that had the estrogen receptor *α* bound. This led to full enhancer activation and efficient transcription of the respective genes [19]. Furthermore, Zhang et al. [54] reported that condensin I downregulation in chicken DT40 cells caused a misregulation of gene expression underlining its role in transcriptional regulation during interphase. Related findings were reported earlier for* C. elegans* where condensins were found at tRNA genes, promoters, and enhancers in interphase, and condensin II binding was associated with a repressive effect on transcription [55]. By contrast, in mouse embryonic stem cells, condensin II and cohesin were present at transcriptional elements of active genes during interphase and affected gene activity in a positive way [56].

In summary, we report a novel interaction partner of Nesprin-2 giant and show that the Nesprin-2 condensin interaction has an impact on mitotic chromosomes. The tight packaging of chromosomes during mitosis, to which the Nesprin-2 interaction might contribute, ensures their faithful segregation and allows them to withstand forces during segregation. Malfunctions in this process can cause DNA bridges which result in chromosome segregation errors and lead to micronucleus formation, and can make chromosomes more prone to DNA damage. It could well be that Nesprins and further NE proteins contribute to this chromosome phenotype. Therefore, mutations in these proteins have the potential to contribute to the formation of distinct clinical manifestations associated with condensin linked diseases [57]. Furthermore, since the Nesprin-2 condensin interaction also takes place during other phases of the cell cycle and since condensins have additional functions in interphase, the Nesprin-2 condensin complex could also affect these processes.

## Supplementary Materials

Supplementary 1 Figure S1: (a) analysis of Nesprin-2 SMC by gel filtration chromatography followed by SDS-PAGE. The elution profile of the Nesprin-2-SMC polypeptide and ovalbumin is shown. (b) GST-Nesprin-2-SMC pulls down Nesprin-2 giant from HaCaT whole cell lysates. Several Nesprin-2 polypeptides derived from the 6885 amino acids protein were identified by mass spectrometry. Amino acid positions are given at the beginning and end of the identified sequences and refer to human Nesprin-2 giant (NCBI accession number: AF435011.1).

Supplementary 2 Figure S2: (a) Nesprin-2 giant is no longer detected by mAb K81-116-6 in lysates from HaCaT cells treated with shRNA directed against the SMC domain and the N-terminus of Nesprin-2. Whole cell lysates from cells treated with the indicated knockdown plasmids were separated in a gradient gel (3 to 12% acrylamide) and probed with mAb K81-116-6. Ne-2 ctrl KD corresponds to a scrambled SMC oligonucleotide. (b) SMC1 and SMC3 do not interact with GST-Nesprin-2-SMC. HaCaT cell lysates (input) were used for precipitation experiments employing GST, GST-Nesprin-2-SMC, and Glutathione-Sepharose beads, respectively, as indicated above the panels. Proteins were separated by SDS-PAGE (10% acrylamide) and the resulting western blots were probed with the antibodies indicated on the right. (c) SMC2 and SMC4 protein levels are not affected in Nesprin-2 knockdown cells. Whole cell lysates from cells treated with the indicated knockdown plasmids were separated by SDS-PAGE (10% acrylamide) and probed for SMC2 and SMC4. Lamin B1 served as control.

Supplementary 3 Figure S3: colocalization of Nesprin-2 and an ER marker in mitotic cells. HaCaT cells were stained with pAbK1 for Nesprin-2 and with protein disulfide isomerase (PDI) specific monoclonal antibodies as ER marker. DNA was stained with DAPI.

Supplementary 4 Figure S4: Nesprin-2 distribution during mitosis. HaCaT cells were labeled with pAbK1, mAb YL1/2 specific for *α*-tubulin, and DAPI for DNA. Bar, 5 *μ*m.

Supplementary 5 Figure S5: specificity of Nesprin-2 association with chromosomes in mitosis. HaCaT cells were stained with pAbK1 for Nesprin-2 and mAb K43-322-2 for Nesprin-1. Bar, 5 *μ*m.

Supplementary 6 Figure S6: (a) proliferation of Nesprin-2-SMC knockdown HaCaT cells. The mean of two independent experiments is shown. (b) Cell cycle progression is unaffected by the loss of Nesprin-2. The experiment was carried out for HaCaT control cells, Nesprin-2-SMC knockdown (Ne-2-SMC KD), and cells treated with a control plasmid containing scrambled sequences. The data show the mean of three independent experiments. No significant differences were noted. M, mitosis; S, S phase; G0/G1, G0, G1 phase.
